# Flexible ureteroscopy with ultrasound guidance for the treatment of parapelvic renal cysts: A complementary approach for locating the cystic wall

**DOI:** 10.1186/s12894-022-00960-6

**Published:** 2022-01-24

**Authors:** Kun-Wu Yan, Xiao-Fei Tian, Na Meng, Wen-Zhan Liu, Zhi-Min Lu, Ming-Tao Guo, Bo Xiao

**Affiliations:** Department of Urology, Handan First Hospital, 25, Cong Tai Road, Handan, 056000 Hebei Province China

**Keywords:** Ultrasound, Flexible ureteroscopy, Parapelvic cysts, Cystic wall

## Abstract

**Background:**

Flexible ureteroscopic incision and drainage is a relatively new surgical method for treating parapelvic cysts. Considering that the intraoperative localization of the cyst may fail with a flexible ureteroscope, we use an innovative ultrasound-guided method to locate the cystic wall during flexible ureteroscopic surgery.

**Methods:**

We retrospectively reviewed 17 consecutive cases of parapelvic renal cysts treated by ultrasound-guided flexible ureteroscopy between March 2017 and May 2020. The differences between the simple flexible ureteroscopic technique and ultrasound-guided flexible ureteroscopic technique were compared. The surgical procedures, postoperative complications, results and patient follow-ups were evaluated.

**Results:**

The cyst wall was seen clearly in 10 patients with ureteroscopic vision. Another 7 patients underwent ultrasound-guided flexible ureteroscopic surgery since it was difficult to identify the cyst wall. The mean operative time was 25.9 ± 8.7 min and 37.1 ± 10.1 min for the conventional and modified techniques, respectively (P = 0.004); the mean time to search for cysts was 17.6 ± 5.8 min and 26.5 ± 8.4 min, respectively (P = 0.002); and the mean incision time was 7.1 ± 4.9 min and 12.1 ± 5.6 min, respectively (P = 0.000). All of the patients were followed-up for 12 months, and no serious complications or recurrence were observed.

**Conclusions:**

We demonstrated that it is feasible and safe to treat parapelvic renal cysts by ultrasound-guided flexible ureteroscopic incision and drainage. The small sample size and need for further studies were the limitations of our work.

**Supplementary Information:**

The online version contains supplementary material available at 10.1186/s12894-022-00960-6.

Parapelvic renal cysts are a special type of renal cystic disease. The prevalence of parapelvic renal cysts is 1–3% among all cases of renal cystic disease [1], and the diameter of these cysts increases with age, especially in 50–70-year-old individuals. Some studies have reported that the occurrence rate is equally common in males and females [2]. This disease often occurs in the unilateral kidney, and the course of disease is slow. Patients with cysts less than 3 cm in diameter mostly have no obvious symptoms or complications and can be monitored with regular follow-up. If the cyst is larger than 3 cm in diameter or if infection, bleeding, or malignancy is suspected, active surgery is indicated. Diagnosing a parapelvic renal cyst by an enhanced computed tomography (CT) scan has an accuracy rate of 80–95% [3]. CT scan criteria can be used to differentiate benign cysts (Type I-II) from malignant cysts (Type III-IV) according to the Bosniak classification [4].

Several techniques are used to treat parapelvic renal cysts. Currently available management options for parapelvic cysts include percutaneous nephroscopic ablation, laparoscopic cyst decortication and flexible ureteroscopy [5]. Ureteroscopic internal drainage of parapelvic cysts is a relatively new procedure. Compared with the other options, the flexible ureteroscope approach has the advantages of a minimally invasive nature, a low complication rate and a short hospital stay [6]. In 2009, Basiri et al. reported the first intrarenal cyst incision and drainage [7]. The clinical feasibility of flexible ureteroscopic management for parapelvic renal cysts has been confirmed in a larger number of patients, but few studies have investigated the localization of parapelvic cysts during endoscopic surgery. To the best of our knowledge, more challenging procedures may be needed if the parapelvic cyst wall is not found during surgery, especially if the cyst wall does not normally bulge into the collection system or the tissue thickness between the collection system and the cyst.

To locate an endoscopic cyst and thereby decrease the need for complicated surgical interventions when an initial attempt to find the cyst wall by direct observation fails, we perform ultrasound-guided flexible ureteroscopy in the treatment of parapelvic cysts. Here, we describe this process for localizing parapelvic cysts and summarize our initial clinical experience.

## Methods

### Patient selection and evaluation

From March 2017 to May 2020, 17 patients with parapelvic renal cysts were admitted to Handan First Hospital. This study retrospectively analyzed clinical data from ultrasound-guided flexible ureteroscopy in the treatment of parapelvic cyst. Informed consent was obtained throughout the process. All of the patients underwent imaging evaluations, including plain films of the kidneys, ureters and bladder (KUB), renal ultrasonography and CT and computed tomography urography (CTU) scans, to define the collecting system anatomy.

The inclusion criteria were as follows: (1) patients with a Bosniak classification on CT imaging of grade I and II; (2) patients with a parapelvic cyst larger than 3 cm in size; (3) patients with urinary obstruction and hydronephrosis caused by a parapelvic cyst compressing the renal calyx or renal pelvis; (4) patients with flank pain, hemorrhage and some other complications caused by a parapelvic cyst; and (5) patients with secondary renal calculi larger than 5 mm in size.

The exclusion criteria were as follows: (1) patients with a Bosniak classification on CT imaging of grade III and IV; (2) patients thought to have a severe urinary tract infection; (3) patients with ureteral stricture; and (4) patients with a history of cardiopulmonary insufficiency.

### Surgical technique

A 6Fr Double-J stent (Laekna, Shanghai, China) was placed two weeks before surgery to dilate the ureter. Routine urine tests and urine cultures were performed, patients with urinary tract infections found before surgery were given oral antibiotic treatment, routine urine tests were rechecked after 3 days, and surgery was performed after the urinary tract infection was effectively controlled. For patients with a history of hypertension before surgery, blood pressure was controlled below 140/90 mmHg by oral antihypertensive drugs. For patients with a history of diabetes, the blood glucose level 2 h after meals was adjusted to less than 8 mmol/l by adjusting the subcutaneous insulin dosage.

The patient was placed in the lithotomy position, routine disinfection of the perineum followed by spreading of towels after general anesthesia was performed, and preplaced 6Fr Double-J stenting was removed by rigid ureteroscopy (Richard Wolf, Germany). The operator cannulated the ureteral orifice with a hydrophilic guidewire (Cook® Medical, Bloomington, IN, USA) into the renal pelvis. Intraoperative ultrasound can be used to show hyperechoic guidewires entering the renal pelvis. The guidewire placement in the renal pelvis was confirmed by ultrasound, and the relevant ureter was routinely examined by rigid ureteroscopy along the guide wire. A ureteral access sheath (Cook® Medical, Bloomington, IN, USA) was inserted into the ureteropelvic junction to facilitate flexible ureteroscopy. The operator surveyed the renal pelvis and calyces sequentially using a digital flexible ureteroscope (Olympus, Tokyo, Japan) to locate the parapelvic cyst wall. In addition, renal stones were handled first if the cyst was combined with calculus, and the renal stones were fragmented to less than 3 mm with a holmium laser (Raykeen, Shanghai, China), which was also used for incisions. The large fragments were removed with a stone basket (Bard, Georgia, USA) to prevent the fragments from entering the cystic cavity after the wall was opened.

Initially, under ureteroscopy, the parapelvic cyst appeared transparent with blue areas when we tried to search for the cyst wall by direct visualization. The renal calyces were chosen as the best incision point, and the renal pelvis was the second choice. On the one hand, the incision site in the calyces was preferred because it is far from the renal hilum, which may damage the peripheral vessels but reduce the incidence of injury to the great vessels. On the other hand, this site is far from the pelvic-ureteral junction, where the high temperature generated by the holmium laser rarely spreads to the ureter, thus reducing the incidence of postoperative ureteral stenosis. A holmium laser was used to make a circular incision from the center of the cyst to the surrounding area; the parameters of the holmium laser were set at 0.8–1.0 J/25–35 Hz; and the diameter of the window was 1.5–2.0 cm so that the cyst and the collecting system could be connected.

**Fig. 1:**
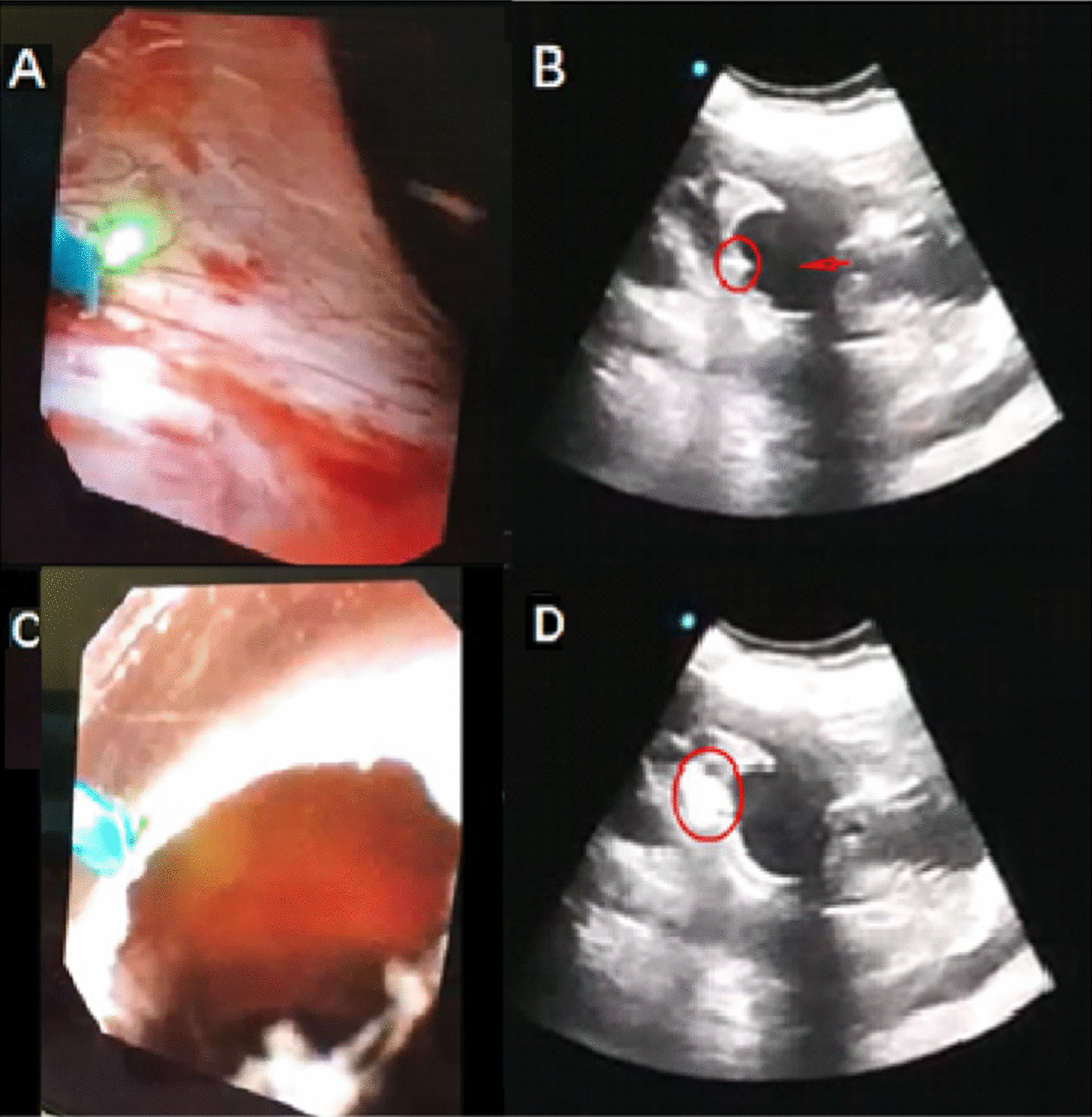
Surgical procedures of ultrasound-guided flexible ureteroscopy. **A** The operator approached the wall of the suspected cyst under a flexible ureteroscopic view and palpated the mucosa in this area. **B** Assistants applied ultrasound to monitor ureteral flexible scopes for contact and compression of the cyst (the arrow points to the cyst, and the rounded area is the end of the flexible ureteroscope). **C** The operator used a laser to cut in the area where the cyst was identified. **D** Assistants used ultrasound to monitor the incision of a parapelvic cyst (the circular area has a smoky appearance on the ultrasound image when the laser was working.)

If the typical blue wall was not found, a search for a suspicious wall that protruded into the renal pelvis was carried out, and the flexible ureteroscope was ultrasound-guided close to the suspicious wall in real time (SIUI, Guangzhou, China). Before the holmium laser was triggered for drainage, the operator confirmed that the flexible ureteroscope was pushing against the cyst wall under ultrasound imaging (Video 1). The incision was performed on an appropriate drainage site, and the typical smoking sign was observed on ultrasound (Fig. 1). For drainage, the proximal double-J stent was coiled in the cyst cavity, which was removed 1–3 months later.

All patients were followed up 3, 6 and 12 months later in our outpatient department. Ultrasonography or CT examinations were used to detect the recurrence of parapelvic cysts and residual stones, and treatments that resulted in cysts shrinking to half their original size by imaging examination at 6 months were considered effective [8–10]. In addition, clinically insignificant residual stones were defined as stones less than 4 mm in diameter.

### Statistical analysis

IBM SPSS statistics v26.0. software was used to analyze the extracted data. Quantitative variables were compared by using the t test, and qualitative variables were compared by using the χ2-test. Continuous data are expressed as the mean and range. The K-S test was used to check whether the preoperative and postoperative data conformed to a normal distribution, and a t test was used to verify whether the data conformed to a normal distribution. Otherwise, the rank sum test was used. p < 0.05 was defined as a statistically significant difference.

## Results

A total of 17 patients, namely, 8 males and 9 females, with parapelvic renal cysts received endoscopic management by flexible ureteroscopy. All operations were conducted successfully, and no patients received open management during surgery. The mean patient age was 52.8 ± 15.1 years (range 45–79 years). There were 6 cases of simple parapelvic cysts and 11 cases of parapelvic cysts with ipsilateral renal calculi. The mean sizes of the cysts and stones were 53.6 ± 7.8 mm (range 40–65 mm) and 10.1 ± 1.7 mm (range 8–12 mm) on preoperative CT scans, respectively (Table 1).

**Table 1:** Seventeen patients’ clinical characteristics, stratified by techniques

	Overall (N = 17)	Simple flexible ureteroscopy (N = 10)	Ultrasound-guided flexible ureteroscopy (N = 7)	*P* value
Age (Years), mean ± SD		52.8 ± 15.1	54.7 ± 15.8	50.3 ± 17.6
Sex, n (%)				
Male		8 (47.1)	5 (50.0)	3 (42.9)
Female		9 (52.9)	5 (50.0)	4 (57.1)
Preoperative symptoms, n (%)				
Pain		4 (23.5)	3 (30.0)	1 (14.3)
Hematuria		2 (11.8)	1 (10.0)	1 (14.3)
Asymptomatic		11 (64.7)	6 (60.0)	5 (71.4)
Mean cyst size (mm), mean ± SD		53.6 ± 5.8	58.5 ± 3.5	53.7 ± 4.9
Locations of the parapelvic cysts, n (%)				
Upper pole		7 (41.2)	4 (40.0)	3 (42.9)
Middle pole		2 (11.8)	0 (00.0)	2 (28.6)
Lower pole		8 (47.1)	6 (60.0)	2 (28.6)
Combined with renal stone, n (%)				
Yes		11 (68.8)	8 (80.0)	3 (42.9)
No		6 (31.2)	2 (20.0)	4 (57.1)
Stone size (mm), mean ± SD		10.1 ± 1.7	14.5 ± 3.2	8.9 ± 2.6

Continuous variables and categorical variables are expressed as the mean ± standard deviation and n (%), respectively

During marsupialization, 10 patients underwent endoscopic management by simple flexible ureteroscopy, and 7 patients were transitioned to ultrasound-guided flexible ureteroscopy because of difficulty locating the cyst wall. The mean operative times of simple flexible ureteroscopy and ultrasound-guided flexible ureteroscopy were 25.9 ± 8.7 min and 37.1 ± 10.1 min, respectively. No severe postoperative complications (such as massive hemorrhage or renal perforation) were observed. Postoperative fever (> 38.5 °C) occurred in 2 patients, backache occurred in 4 patients, and the clinical symptoms were alleviated 3–5 days after surgery. The 12-month follow-up showed that 12 cysts became undetectable, while 5 cysts decreased in size by at least half (Table 2).

**Table 2:** Intraoperative data, postoperative values and procedural outcomes

	Overall (N = 17)	Simple flexible ureteroscopy (N = 10)	Ultrasound-guided flexible ureteroscopy (N = 7)	*P* Value
Mean operative time (min), mean ± SD	30.8 ± 8.4	25.9 ± 8.7	37.1 ± 10.1	0.004
Searching for the renal cyst time (min), mean ± SD	22.7 ± 5.3	17.6 ± 5.8	26.5 ± 8.4	0.002
Incision of the renal cyst time (min), mean ± SD	10.8 ± 4.7	7.1 ± 4.9	12.1 ± 5.6	0.000
Hospitalization time (days), mean ± SD	3.0 ± 1.2	2.6 ± 1.5	3.4 ± 1.7	0.001
Postoperative complications, n(%)				0.540
Fever	2 (12.5)	1 (10.0)	1 (14.3)	
Backache	4 (25.0)	1 (10.0)	3 (42.9)	
Sepsis	0 (0)	0 (0)	0 (0)	
Follow-up of one year, n(%)				0.949
Disappearance rate of the cyst	12 (70.6)	7 (70.0)	5 (71.4)	
Regression rate of the cyst	5 (29.4)	3 (30.0)	2 (28.6)	

Continuous variables and categorical variables are expressed as the mean ± standard deviation and n (%), respectively

## Discussion

Renal cysts are a common cystic disease that occur in 5% of cysts in the general population, and most of them do not require any treatment [11]. Parapelvic renal cysts are rare renal cysts adjacent to the collecting system and the vessels of the renal hilum. The ratio of men to women with parapelvic renal cysts is similar; most patients are older than 50, and age is directly related to an increase in cyst diameter [12]. However, parapelvic cysts may cause clinical symptoms earlier than simple renal cysts and are more frequently associated with pain, hematuria, infection, hypertension, hydronephrosis and stone formation [13–15]. Some scholars have concluded that small cysts (≤ 4 cm in diameter) without obvious symptoms or complications and without obvious pelvic compression on imaging can be monitored with regular follow-up [16]. Cysts larger than 4 cm in diameter with symptoms of compression and suspected infection and bleeding should be treated surgically. Other scholars have suggested that surgery should be considered if the cyst is larger than 2 cm in diameter and has clinical symptoms or comorbidities [17]. The purpose of surgery is to drain the contents of the cyst to prevent further compression of the kidney by cystic fluid, remove stones, and correct infection caused by obstructive factors. Thus, earlier surgical intervention is required for parapelvic renal cysts than for simple renal cysts.

To date, some studies have reported flexible ureteroscopic treatment of this disease and have shown the technique to be feasible and safe in selected patients. Compared with percutaneous resection or ablation, the work access sites associated with percutaneous nephrostomy (PCN) inevitably cause renal injury, such as severe perinephritis, retroperitoneal abscess and secondary ureteropelvic junction obstruction. From 29 to 83% of treated patients exhibit complications [18, 19]. Compared with laparoscopic unroofing, the latter has a high risk of injury to the renal cortex and renal pedicle since parapelvic cysts are usually surrounded by renal parenchyma [7, 20]. The retrograde approach may be less invasive for entirely endophytic cysts. The flexible ureteroscopic technique has lowered the risk of serious complications [21].

The location of the renal cyst wall is a crucial step of marsupialization for the treatment of parapelvic cysts. Liaconis et al. reported that the light blue color of the cystic wall under ureteroscopy is helpful for locating cysts [22]. We also found some cases of typical cysts in our study; however, these features were not discovered if the cyst wall was relatively thick. Another study by Zhixian Wang et al. reported methylene blue injection via percutaneous renal cyst puncture to identify parapelvic cysts. This method was successfully used to locate the cystic wall in their research [23]. However, in previous studies [20], we found that the blue cyst wall that was injected with methylene blue was also difficult to identify if the typical capsular wall was not found during surgery. The cystic wall has the same color as other parts of the renal pelvis because it is relatively thick, and it is challenging for the operator to locate the cystic wall and to choose the area for incision.

The primary aim of this study was to present a method for locating cystic walls during routine flexible ureteroscopy when parapelvic cysts could not be identified. Intraoperative ultrasound has been used increasingly in recent years. Ultrasound has the advantages of real-time monitoring of cysts and guided flexible ureteroscopy, which can help in locating cystic walls and adjusting the incision direction. Kang N et al. reported the experience of flexible ureteroscopy combined with ultrasound to search for parapelvic cysts. The holmium laser presented linear high-echo, and cysts presented low-echo under ultrasound imaging [8]. Additionally, the adjacent relationship between the flexible ureteroscope and cyst can be shown under ultrasound imaging, and the best area for incision and inner drainage can be identified. In our study, more than half of the cysts were found under ureteroscopic vision, as we demonstrated success in 10 patients in this study. In cases where the cyst wall had the same color as other parts of the renal pelvis, ultrasound was employed. We found that this technique can eliminate methylene blue injection via percutaneous renal cyst puncture and reduce the patient's pain without prolonging the operation time. From the literature, we found that in patients with internal drainage of parapelvic cysts, a radial incision or circular incision was usually performed, and the diameter of the incision was usually 1–2 cm [9, 10]. To ensure that the cystic cavity is fully connected to the renal pelvis, we usually perform a circular incision of approximately 2 cm in diameter centered on the incision point.

During a mean follow-up period of 14 months (range 12–18 months), ultrasound and CT showed no cyst recurrence. The results suggest that our techniques prevent further compression of the collecting system and promote complete drainage of cystic fluid. We provide an alternative method that can be selected for patients with parapelvic cysts.

Our research has the limitations of a small patient sample since parapelvic cysts are not relatively common. Additionally, the inherent defects of retrospective studies and the lack of long-term follow-up led to flaws in the study. There were few patients who required ultrasound-guided flexible ureteroscopy. In this situation, designing and conducting a randomized controlled trial was difficult, and we chose to perform retrospective research instead.

## Conclusion

Ultrasound guidance is a modified method for treating parapelvic cysts by flexible ureteroscopy. According to our results, this procedure is a feasible, safe, and effective approach for treating parapelvic cysts. Further studies with large samples and longer follow-ups need to be performed to assess the long-term efficacy of this procedure.

## Supplementary Information

**Additional file 1.** Demonstration of ultrasound-guided flexible ureteroscope procedure.

## Data Availability

The datasets used and/or analysed during the current study are available from the corresponding author on reasonable request.

## Abbreviations

No: Number
CT: Computed tomography
PCN: Percutaneous nephrostomy
KUB: Plain films of the kidneys, ureters and bladder
min: Minute
mm: Millimeter
SD: Standard deviation
